# Case-based learning and multiple choice questioning methods favored by students

**DOI:** 10.1186/s12909-016-0564-x

**Published:** 2016-02-02

**Authors:** Magalie Chéron, Mirlinda Ademi, Felix Kraft, Henriette Löffler-Stastka

**Affiliations:** Department for Psychoanalysis and Psychotherapy, Advanced Postgraduate Program for Psychotherapy Research, Medical University of Vienna, Waehringer Guertel 18-20, 1090 Vienna, Austria; Research Center for Molecular Medicine of the Austrian Academy of Sciences, Vienna, Austria; Department of Anaesthesia, General Intensive Care and Pain Therapy, Medical University Vienna, Vienna, Austria

**Keywords:** Case-based questioning, Learning process, Questions’ formulation, Medical education, Curriculum, Assessment, Multiple Choice Question

## Abstract

**Background:**

Investigating and understanding how students learn on their own is essential to effective teaching, but studies are rarely conducted in this context. A major aim within medical education is to foster procedural knowledge. It is known that case-based questioning exercises drive the learning process, but the way students deal with these exercises is explored little.

**Methods:**

This study examined how medical students deal with case-based questioning by evaluating 426 case-related questions created by 79 fourth-year medical students. The subjects covered by the questions, the level of the questions (equivalent to United States Medical Licensing Examination Steps 1 and 2), and the proportion of positively and negatively formulated questions were examined, as well as the number of right and wrong answer choices, in correlation to the formulation of the question.

**Results:**

The evaluated case-based questions’ level matched the United States Medical Licensing Examination Step 1 level. The students were more confident with items aiming on diagnosis, did not reject negatively formulated questions and tended to prefer handling with right content, while keeping wrong content to a minimum.

**Conclusion:**

These results should be taken into consideration for the formulation of case-based questioning exercises in the future and encourage the development of bedside teaching in order to foster the acquisition of associative and procedural knowledge, especially clinical reasoning and therapy-oriented thinking.

## Background

Trying to understand how students learn on their own, aside from lectures, is essential to effective teaching. It is known that assessment and case-based questioning drive the learning process. Studies have shown that the way assessment is being conducted influences students’ approach to learning critically [1]. Several written methods are used for the assessment of medical competence: Multiple Choice Questions (MCQs), Key Feature Questions, Short Answer Questions, Essay Questions and Modified Essay Questions [2]. Based upon their structure and quality, examination questions can be subdivided into (1) open-ended or multiple choice and (2) context rich or context poor ones [3, 4].

Well-formulated MCQs assess cognitive, affective and psychomotoric domains and are preferred over other methods because they ensure objective assessment, minimal effect of the examiner’s bias, comparability and cover a wide range of subjects [5]. Context rich MCQs encourage complex cognitive clinical thinking, while context poor or context free questions mainly test declarative knowledge (facts, “what” information), which involves pure recall of isolated pieces of information such as definitions or terminologies. In contrast, procedural knowledge (“why” and “how” information) requires different skills: Students are encouraged to understand concepts and to gather information from various disciplines in order to apply their knowledge in a clinically-oriented context. Remarkably, prior clinical experience has been suggested to be a strong factor influencing students’ performance in procedural knowledge tasks [6, 7]. With the focus of teaching students to think critically, test items must require students to use a high level of cognitive processing [3]. A successful approach is using Extended Matching Items (EMIs), consisting of clinical vignettes [2]. This format is characteristic for examination questions in Step 2 of the United States Medical Licensing Examination (USMLE) [8]. Step 2 items, test the application of clinical knowledge required by a general physician and encourage examinees to make clinical decision rather than to simply recall isolated facts [6]. As a well-established examination format introduced by the *National Board of Medical Examiners* (NBME) USMLE question criteria served for the comparison in our study.

Case-based learning (CBL) has gained in importance within past years. This well-established pedagogical method has been used by the Harvard Business School since 1920 [9]. Nevertheless, there is no international consensus on its definition. CBL as introduced to students of the Medical University of Vienna (MUV, Austria) in *Block 20* is inquiry-based learning demanding students to develop clinical reasoning by solving authentic clinical cases presented as context rich MCQs. Generally, exposing students to complex clinical cases promotes (1) self-directed learning, (2) clinical reasoning, (3) clinical problem-solving and (4) decision making [9]. In contrast to other testing formats CBL facilitates deeper conceptual understanding. As students see the direct relevance of the information to be learnt, their motivation increases and they are more likely to remember facts. Studies showed that CBL fosters more active and collaborative learners and that students enjoy CBL as a teaching method [9]. This is in line with the improving results from the students’ evaluation of the course *Psychic Functions in Health and Illness and Medical Communication Skills-C* (*Block 20/ÄGF-C*) performed in 2013 [10] and 2014 [11] at the MUV. A Likert scale ranging from 1 (very bad) to 4 (very good) was used to evaluate the quality of the lectures: The mean grade improved from 1.6 in 2013 to 3 one year later, after the introduction of online case-based exercises related to the lectures. Therefore, based upon the assumption that students tend to prefer practical learning, the development of case-based question driven blended-learning will be further encouraged at the MUV, in order to aim for an effective training for the fostering of procedural knowledge, necessary for clinical reasoning processes and clinical authentic care.

There are no published studies analyzing the way medical students construct MCQs regarding their level of clinical reasoning. To learn more about how students deal with case-based questioning we analyzed student-generated MCQs. The study gives important insights by examining students’ way of reasoning, from formal reasoning using only declarative knowledge to clinical and procedural reasoning based on patients’ cases. Moreover, this study allows to observe to what extent negatively formulated questions, a rarely used format in exams, may not be a problem for students.

## Methods

This analysis is based on the evaluation of a compensatory exercise for missed seminars completed by students in their fourth year of medical studies at the MUV, after attending their first course on psychic functioning (*Block 20/ÄGF-C*). The 5-week-long *Block 20* [12] focused on the fundamentals of psychic functions, the presentation of the most important psychological schools and on the significance of genetic, biological, gender-related and social factors, as well as on the presentation of psychotherapeutic options and prevention of psychic burden [13]. Basis of the doctor-patient communication and of psychological exploration techniques were offered [12]. To pass *Block 20*, all students had to take part to the related online CBL [14] exercise. This exercise presented patients cases including detailed information on diagnosis and therapy, subdivided in psychotherapy and pharmacology. The students had to answer MCQ concerning each diagnostic and therapeutic step.

The current study was approved by the ethics committee of the Medical University of Vienna, students gave informed consent to take part and data is deposited in publicly available repositories (online CBL exercise) after finishing the study. The students were instructed to create MCQs with 4–5 answer possibilities per question, related to cases of patients with psychopathological disorders presented in the online CBL exercise and in the lectures’ textbook of the *Block 20* [15]. The “One-Best Answer” format was recommended. Additionally, the students were required to explain why the answers were right or wrong. MCQ examples were offered to the students in the online CBL exercise.

The authors performed the assessment and classification of the students’ MCQs after group briefings. A final review was done by MC to ensure inter-rater reliability, it was stable at *k* = .73 between MC and HLS.

### Subjects covered by the questions

The proportions of epidemiology, etiology/pathogenesis, diagnosis and therapy oriented items were examined. In order to simplify the classification, etiology and pathogenesis items were gathered into one group. Items asking for symptoms, classifications (e.g. ICD 10 criteria) as well as necessary questions in the anamnesis were gathered as diagnosis items. Among the therapy items, the frequency of items concerning psychotherapy methods and pharmacology was also compared. The proportion of exercises including at least one diagnosis item and one therapy item was observed. These subjects were chosen according to the patient cases of the CBL exercises and the lectures’ textbook.

### Level of the questions

The level of the questions was evaluated in comparison to USMLE equivalent Steps 1 and 2, as described by the NBME. While Step 1 questions (called *recall items*) test basic science knowledge, “every item on Step 2 provides a patient vignette” and tests higher skills. Step 2 questions are necessarily *application of knowledge items* and require interpretation from the student [6].

To assess the level of the items, 2 further groups were created, distinguishing items from the others. Examples of Step 1 and Step 2 Items’ stems offered by the students:

Step 1: “What is the pharmacological first line therapy of borderline patients?”

(Item 31)

Step 2: “M. Schlüssel presents himself with several medical reports from 5 specialists for neurology, orthopedics, trauma surgery, neuroradiology and anesthesia, as well as from 4 different general practitioners. Diagnostic findings showed no evidence for any pathology. Which therapy options could help the patient?” (Item 86)

Further, “Elaborate Items” were defined by the authors as well thought-out questions with detailed answer possibilities and/or extensive explanations of the answers.

Finally, “One-Step questions” and “n-steps questions” were differentiated. This categorization reflects the number of cognitive processes needed to answer a question and estimates the complexity of association of a MCQ. *Recall items* are necessarily one-step questions, whereas *application of knowledge items* may be one-step questions or multiple (n) steps questions. Because the evaluation of the cognitive processes is dependent on the knowledge of the examinee, the NBME does not give priority to this categorization anymore, although it gives information on the level and quality of the questions [6]. The previous example of a Step 2 question (Item 86) is an *application of knowledge* (Step 2) item categorized as n-Step item, because answering the question necessitates an association to the diagnosis, which is not explicitly given by the question. An example for a Step 2 question necessitating only one cognitive process would be: “A patient complains about tremor and excessive sweating. Which anamnestic questions are necessary to ask to diagnose an alcohol withdrawal syndrome?” (Item 232)

### Formulation of the questions

The proportion of positively and negatively formulated questions created by the students was examined, as well as the number of right and wrong answer choices, in correlation to the formulation of the question.

### Statistics

Descriptive statistics were performed using SPSS 22.0 to analyze the subjects covered by the questions, their level and formulation, and the number of answer possibilities offered. The significance of the differences was performed using the Chi-square Test or Mann–Whitney U Test, depending on the examined variable, after testing for normal distribution. A given *p*-value < .05 was considered statistically significant in all calculations.

## Results

The study included 105 compensation exercises, performed by 79 students, representing 428 MCQs. After reviewing by the examiner (HLS), who is responsible for pass/fail decisions on the completion and graduation concerning the curriculum element Block 20/ÄGF-C, followed by corrections from the students, two questions were excluded, because the answers offered were not corresponding to the MCQ’s stem. Finally, 426 questions remained and were analyzed.

### Subjects covered by the questions

The subjects covered by the 426 questions concerned the diagnosis of psychiatric diseases (49.1 %), their therapies (29.6 %) and their etiology and pathogenesis (21.4 %). 18 questions covered two subjects (Table 1).

**Table 1:** Question characteristics: subjects, level and formulation

			number	percent
Students			79	
Compensation Exercises			105	
Questions		valid	426	
		excluded	2	
Subjects covered by the questions				
Epidemiology		all	18	4.2
		Step 1	18	4.2
		Step 2	0	0
Etiology/Pathogenesis		all	91	21.4
		Step 1	89	20.9
		Step 2	2	0.5
Diagnosis (Symptoms, Classifications…)		all	209	49.1
		Step 1	190	44.6
		Step 2	19	4.5
Therapy	Psychotherapy	all	52	12.2
		Step 1	48	11.3
		Step 2	4	0.9
	Pharmacology	all	74	17.4
		Step 1	66	15.5
		Step 2	8	1.9
Level of the questions				
Step 1 questions			395	92.7
Step 2 questions			31	7.3
Elaborate questions and/or answers			199	46.7
One-Step questions			421	98.8
n-Steps questions			5	1.2
Formulation of the questions				
Positively formulated			309	72.5
Negatively formulated			117	27.5

Significantly more items concerned the diagnosis of psychiatric diseases than their therapies (*p* < .001, Chi-Square Test); 63.3 % of the students offered at least one item regarding diagnosis and one item regarding therapy in their exercise.

Among the therapy items, significantly more pharmacology items were offered than psychotherapy items (59 % versus 41 %; *p* = .043, Chi-Square Test).

### Level of the questions

395 (92.7 %) of the questions were classified as Step 1-questions. Nevertheless, 199 (46.7 %) of the questions were elaborate. 421 (98.8 %) out of the 426 questions were One-Step questions, according to USMLE criteria (Table 1). From the 18 questions covering two subjects, 16 were Step 1-questions.

### Formulation of the questions

72.5 % of the questions were positively formulated, 27.5 % negatively. A significant difference was observed between the positively and negatively formulated questions regarding the number of right answers: Table 2 shows the distribution of the number of right answers, depending on the questions’ formulation. The students offered significantly more right answer possibilities per positive-formulated question than per negative-formulated questions (*p* < .001, Mann–Whitney U Test).

**Table 2:** Questions’ characteristics depending on the questions’ formulation

Number of right answers depending on the questions’ formulation							
		Question formulation					
		Negative		Positive		Total	
		n	%	n	%	n	%
Number of right answers							
	1	112	95.7	196	63.4	308	72.3
	2	5	4,3	31	10,0	36	8,5
	3	0	0	49	15,9	49	11,5
	4	0	0	28	9,1	28	6,6
	5	0	0	5	1,6	5	1,2
Total		117	100	309	100	426	100
Mean ± SD		1.04 ± 0.2*		1.75 ± 1.1*			
Total number of answers depending on the questions’ formulation							
Number of answers							
	1	0	0	6	1,9	6	1,4
	2	0	0	4	1,3	4	0,9
	3	0	0	6	1,9	6	1,4
	4	30	25,6	138	44,7	168	39,4
	5	87	74,4	152	49,2	239	56,1
	6	0	0	2	0,6	2	0,5
	7	0	0	1	0,3	1	0,2
Total		117	100	309	100	426	100
Mean ± SD		4.74 ± 0.44^*^		4.41 ± 0.79^*^			
Number of elaborate questions depending on the questions’ formulation							
		Question formulation					
		Negative		Positive		Total	
		n	%^(1)^	n	%^(1)^	n	%^(1)^
Elaborate question							
	No	68	58.1	159	51.5	227	53.5
	Yes	49	41.9	150	48.5	199	46.5
Total		117	100	309	100	426	100

*p < 0.001 (Mann–Whitney U Test); SD: Standard deviation; Mean and SD refer to the number of right answers per question and the number of answers per question, respectively, depending on the formulation of the questions; (1) % within formulation groups

The positive-formulated questions had more often two or more right answers than the negative-formulated questions (*p* < .001, Chi-square value = 44.2).

The students also offered less answer possibilities per positive-formulated question than per negative-formulated question (*p* < .001, Mann–Whitney U Test). Further, Table 2 presents the distribution of the number of answers offered depending on the questions’ formulation. The proportion of questions with 4 answer possibilities instead of 5 is higher within the group of positive-formulated questions (*p* < .001, Chi-square value = 16.56). Regarding the proportion of elaborate questions depending on their formulation, there was no significant difference.

Twenty-nine (36.7 %) students offered only positively formulated questions. The students who formulated at least one question negatively (63.3 %) formulated 41.1 ± 22.4 % of their questions negatively.

## Discussion

### Many more questions aiming on diagnosis

At the end of year 4, students of the MUV had had various lectures but hardly any actual experiences with therapies. This may explain why significantly more items concerned the diagnosis of psychiatric diseases than their therapies.

### Among questions aiming on therapy, significantly more concerned pharmacotherapy than psychotherapy

Before *Block 20*, the seminars concerning therapies in the MUV Curriculum were almost exclusively pharmacological. After successful attendance of *Block 20* most students who did not have any personal experience of psychotherapy only had little insight into how psychotherapy is developing on the long-term and what psychotherapy can really provide to the patient. Psychotherapy associations were still loaded with old stereotypes [13, 16]. This could explain why significantly more therapy questions addressed pharmacology than psychotherapy.

### A huge majority of Step 1 questions

The students mainly offered Step 1 questions. It can be questioned, whether the lack of case-oriented questions was an indication for insufficient clinical thinking by the students. An essential explanation could be that students lacked adequate patient contact until the end of year four. Indeed, MUV students were allowed to begin their practical experience after year two and eight compulsory clerkship weeks were scheduled before the beginning of year five [17]. Thus, Austrian medical students gained consistent clinical experience only after year four, with rotations in year five and the newly introduced *Clinical Practical Year* in year six. A European comparison of medical universities’ curricula showed that students of other countries spent earlier more time with patients: Dutch, French and German medical students began with a nursing training in year one and had 40, 10 and 4 months, respectively, more clerkship experience than Austrian students before entering year five [18–21]. French and Dutch universities are extremely centered on clinical thinking, with a total of 36 clerkship months in France and the weekly presence of patients from the first lectures on in Groningen [22]. Thus, it would be interesting to repeat a similar case-based exercise in these countries to explore if medical students at the same educational stage but with more practical experience are more likely to offer patient vignette items.

### Students preferred to work with right facts and did not reject negatively worded questions

As negatively worded questions were usually banished from MCQ exams, it was interesting to observe that medical students did not reject them. In fact, negatively formulated questions are more likely to be misunderstood. Their understanding correlates to reading ability [23] and concentration. Although many guidelines [6, 24] clearly advised to avoid negative items, the students generated 27.5 % of negatively formulated questions. Also *Pick N format*-questions with several right answers were offered by the students, despite the recommendations for this exercise: They offered significantly less total answer possibilities but significantly more right answers to positively worded questions than to negatively worded questions. Those results supported the hypothesis that the students preferred handling right content while keeping wrong content to a minimum.

Several possible reasons can be contemplated. When students lack confidence with a theme and try to avoid unsuitable answer possibilities, it can be more difficult to find four wrong answers to a positively worded question instead of several right answers, which may be listed in a book. Furthermore, some students may fear to think up wrong facts to avoid learning wrong content. Indeed, among positively worded items, 26.6 % were offered with 3 or more right answers, which never happened for negatively worded items (Table 2).

Notably, “right answer possibilities” of negatively worded items’ stems as well as “wrong answer possibilities” of positively worded items’ stems are actually “wrong facts”. For example, the right answer of the item “Which of the following symptoms does NOT belong to ICD-10 criteria of depression?” (Item 177) is the only “wrong fact” of the 5 answer possibilities. Writing the 4 “wrong answers” of this question, which are actually the ICD-10 criteria for depression, can help the students learn these diagnostic criteria. On the contrary, the “right answers” to a positively worded item such as “Which vegetative symptoms are related to panic attacks?” (Item 121) are the true facts.

Finally, the students’ interest for right facts supports the theory that a positive approach, positive emotions and curiosity are favorable to learning processes. Indeed, asking for right content is a natural way of learning, already used by children from the very early age. The inborn curiosity — urge to explain the unexpected [25], need to resolve uncertainty [26] or urge to know more [27]— is shown by the amount of questions asked by children [28, 29]. The students’ way to ask for right contents appears very close to this original learning process.

The inputs of developmental psychology, cognitive psychology as well as of neurosciences underline this hypothesis. Bower presented influences of affect on cognitive processes: He showed a powerful effect of people’s mood on their free associations to neutral words and better learning abilities regarding incidents congruent with their mood [30]. Growing neurophysiological knowledge confirmed the close relation between concentration, learning and emotions — basic psychic functions necessitating the same brain structures. The amygdala, connected to major limbic structures (e.g. pre-frontal cortex, hippocampus, ventral striatum), plays a major role in affect regulation as well as in learning processes [15], and the hippocampus, essential to explicit learning, is highly influenced by stress, presenting one of the highest concentrations of glucocorticoid receptors in the brain [31]. Stress diminishes the synaptic plasticity within the hippocampus [32], plasticity which is necessary to long-term memory.

Neuroscientific research also underlined the interdependence of cognitive ability and affect regulation. Salas showed on a patient after an ischemic stroke event with prefrontal cortex damage that, due to executive impairment and increased emotional reactivity, cognitive resources could not allow self-modulation and reappraising of negative affects anymore [33].

Considering this interdependence, right contents might be related to a positive attitude and positive affects among the students. It could be interesting to further research on this relation as well as on the students’ motivations concerning the formulation of the questions.

The combination of those reasons probably explains why the students offered significantly more wrong answers to negatively worded items and more right answers to positively worded items, both resulting in the use of more right facts. All the students’ assessment questions and associated feedback were used to create a new database at the MUV trying to integrate more right facts in case-based learning exercises in the future.

The main limitation concerns the small sample size and the focus on only one curriculum element. Further studies with convenient sampling should include other medical fields and bridge the gap to learning outcome research.

## Conclusion

The evaluation of the questions offered by medical students in their fourth year at the MUV showed that the students were much more confident with items aiming on diagnosis. Among items aiming on therapy, they proved to be more confident with pharmacotherapy than with psychotherapy. These results, together with the improving evaluation of the *Block 20* after introducing CBL exercises and the international awareness that case-based questioning have a positive steering effect on the learning process and foster the acquisition of associative and procedural knowledge, should encourage the further development of affective positively involving case-based exercises, especially with a focus on clinical reasoning and therapy-oriented thinking.

The development of bedside teaching and the implementation of clerkships from the first year of studies (e.g. a 4-week practical nursing training) could also be considered in order to stimulate earlier patient-centered thinking of the students of the MUV. A comparison with the level of clinical reasoning of medical students from countries where more practical experience is scheduled during the first year of study would be interesting.

Concerning assessment methods and particularly the formulation of case-based questions, the students did not reject negatively formulated questions, but showed a tendency to prefer working with right contents, while keeping wrong content to a minimum. This preference could be further explored and considered in the future for the formulation of MCQs in case-based exercises.

## Availability of supporting data

Data of the patients’ cases, on which the MCQs created by the students were based on, can be found in the textbook of the curriculum element and lectures [15] and via the Moodle website of the Medical University of Vienna [34]. The Moodle website is available for students and teachers of the Medical University of Vienna with their username and password. The analyzed and anonymous datasets including the MCQs [34] are accessible on request directly from the authors.

## Abbreviations

CBL: Case-based learning
EMI: Extended Matching Items
MCQ: Multiple Choice Question
MUV: Medical University of Vienna
NBME: National Board of Medical Examiners
USMLE: United States Medical Licensing Examination
